# Molluscicidal activity of *Nicotiana tabacum* extracts on the invasive snail *Pomacea canaliculata*

**DOI:** 10.1038/s41598-023-38141-6

**Published:** 2023-07-18

**Authors:** Jing Guo, Shaobin Zhang, Jian Zeng, Yingtong Chen, Yongxin Guo, Jinling Liu, Ailan He

**Affiliations:** 1 School of Biology and Agriculture, Shaoguan University, Shaoguan, 512005 China; 2 Department of Ecology, College of Natural Resources and Environment, South China Agricultural University, Guangzhou, 510642 China; 3 College of Biology and Food Engineering, Guangdong University of Education, Guangzhou, 510303 China

**Keywords:** Ecology, Ecology

## Abstract

Botanical molluscicides for controlling the invasive snail *Pomacea canaliculata* have attracted worldwide attention because of their cost and environmental friendliness. Aqueous extracts from discarded tobacco leaf (*Nicotiana tobacum*) were evaluated for molluscicidal activity against different-sized *P. canaliculata* under laboratory conditions. The results showed that over 90% of the snails died in 1 g/L tobacco extract within 4 days, and the survival of *P. canaliculata* was inversely proportional to the snail size, tobacco extract concentration and length of exposure time. Adult males were more susceptible to tobacco extract than females. The snails had few chances to feed or mate in 0.5 g/L tobacco extract, and reproduction was greatly limited in 0.2 g/L. The growth of juvenile snails was inhibited in 0.2 g/L tobacco extract, but adults were unaffected. The antioxidant capacity of *P. canaliculata* in response to tobacco extract can be size- and sex-dependent, and the activities of superoxide dismutase, catalase, and acetylcholinesterase and the contents of glutathione and malondialdehyde were increased in adult males. These results suggest that discarded tobacco leaves can be useful as a molluscicide for controlling the invasive snail *P. canaliculata* based on its effects on survival, behaviour, food intake, growth performance and antioxidant capacity.

## Introduction

The golden apple snail *Pomacea canaliculata* (Lamarck 1822) originates from South America and was introduced as a high-protein food source to mainland China four decades ago^1^. A breeding boom in *P. canaliculata* farming occurred in the 1980s, and the snails quickly spread to almost half of China^2^. Due to the unpleasant taste and market failure, the snails were abandoned and entered various wetland habitats. Because of its voracious appetite, fast growth and high reproductive rate, *P. canaliculata* has caused incalculable rice production losses in southern China^2^. Moreover, it is the predominant intermediate host of *Angiostrongylus cantonensis* (Chen 1935), which can lead to eosinophilic meningitis in humans^1,3^. Given the harm and threat of *P. canaliculata* to crops and human health, finding highly effective control strategies for the snail has been a trending research topic in recent years.

Synthetic chemical molluscicides are mainly used to control snails but are toxic to nontarget organisms and cause serious environmental hazards^4^. Many studies have screened hundreds of botanical molluscicides for molluscicidal activity, and some of these have been shown to have high bioactivity in controlling *P. canaliculata*, be easily available, easily biodegradable and safe for humans and the environment^3–6^. However, these botanical molluscicidal materials are not field crops, which creates instability in their supply and high labour costs. *Nicotiana tobacum* L. is one of the most commercially valued crops, and China is the largest producer and consumer of tobacco worldwide. The annual output of tobacco waste is over 460,000 tons during processing and cigarette making in China^7^.

More than 40 alkaloids occur in tobacco, and the nicotine content accounts for over 95%^8^. Nicotine is commonly used as a traditional botanical insecticide, considering that it has strong systemic activity, stomach-poisoning effect and contact toxicity, and it has a strong affinity for the acetylcholine (ACh) receptor, preventing ACh from being properly conducted and further blocking and suffocating nerves^9,10^. A field trial in Thailand showed that 1562.5 kg per hectare of tobacco waste from the cigarette production process could kill all *P. canaliculata* within 2 days^11^.

Moreover, the tobacco waste can be increased rice growth and yield without nicotine inspected in rice grain^11^. However, the components of tobacco waste and their proportion are unclear, and the information available on the effects of tobacco on *P. canaliculata* is limited, which might be difficult for other researchers and farmers to learn and apply. Previous research has shown that the nicotine content of tobacco leaves is much higher than that of roots after topping, and the nicotine content of stems is lowest throughout the development of tobacco plants^12^. During tobacco planting and processing, a large number of discarded tobacco leaves that account for approximately 25% of the total tobacco output are produced, which pollutes the environment and wastes resources^13^. If discarded tobacco leaves can effectively control the damage to *P. canaliculata*, the utilization rate and added value of tobacco leaves will be greatly improved, while the use of synthetic chemical molluscicides can be reduced or avoided. Hence, the present study was carried out to evaluate the potential use of *N. tabacum* leaf extracts on the survival, behaviour, growth and antioxidant capacity of *P. canaliculata*. The information generated from this research could be beneficial for further studies on the efficient use of tobacco waste for green prevention and control of *P. canaliculata*.

## Materials and methods

### Experimental materials

All experimental research was conducted in accordance with relevant institutional, national, and international guidelines and legislation. *Pomacea canaliculata* snails were collected from irrigation ditches in farmland near Shaoguan University, Shaoguan, China (24° 78′ N, 113° 67′ E), and identified as *P. canaliculata* according to the mitochondrial COI gene sequence^14^. The snails were reared in two 135-L rectangular aquaria and fed fresh lettuce *Lactuca sativa* L. under sunlight for approximately 1 week before we used them in this experiment. The tested snails were divided into three classes according to shell height: 5‒15 mm (small), 15‒25 mm (medium) and 25‒40 mm (large). The snails were considered to be sexually mature if their shell heights were greater than 25 mm^15^, and shell height was defined as the horizontal distance between the top and base of the shell^16^. Male snails can be easily distinguished from females once they reach the age of maturity by observing the operculum^17^, so large snails were divided into two groups: adult males and females. Discarded flue-cured tobacco leaf (*N. tabacum* cv. K326) was provided by a farmer who exploits tobacco-rice rotation in Shixing County, Shaoguan City, China (25° 02′ N, 114° 15′ E) that permitted by Tobacco Research Laboratory, South China Agricultural University. And the experiments were performed in accordance with regulations of the Guangdong Tobacco Shaoguan Company. The collected tobacco leaves were dried at 60 °C for 12 h considering their moisture content in storage and then smashed into powder by a grinder before the experiment. Specimen (voucher no. 2021-03-SGSX) was stored at − 20 °C refrigerator in South China Agricultural University (contact person: Huaiyuan Li, email: leehy@scau.edu.cn). We confirm that this complies with national guidelines and no formal ethics approval was required in this case. The nicotine content of tobacco was determined using the spectrometric method (ISO 2881-1992).

### Experimental procedure

Fifty grams of tobacco powder was mixed with 1 L of dechlorinated tap water in a conical flask, and then the flask was kept at 60 °C for 24 h in a water bath. A stock solution of 50 g/L aqueous tobacco extract was prepared after filtration through a two-layer gauze. Different concentrations of tobacco extract were obtained by diluting the stock solution with dechlorinated tap water to 1 and 2 g/L concentrations. Moreover, 0 g/L tobacco extract (dechlorinated tap water) served as the control group (CK). Thirty-six plastic cases were used for the survival study, which were placed in a room. Ten actively crawling *P. canaliculata* of each type (small, medium, adult male, adult female) were placed in each plastic case (25 × 14 × 13 cm) containing 2 L of tobacco extract, and each concentration (0, 1, 2 g/L) was tested in triplicate, so 90 individuals of *P. canaliculata* for each type were used. During the experimental period, the snails were not fed, and water was kept at an average temperature of 25 ± 2 °C. Each case was covered with a perforated lid to prevent the escape of snails. The survival rates of *P. canaliculata* of each type were assessed at 24, 48, 72 and 96 h after they were introduced into the tobacco extracts. The snails were confirmed dead if a rotten smell was detected or the operculum was unable to contract after a light touch^2^. The dead snails were removed from the cases immediately once found to avoid water pollution.

The trial found that the snails were short-lived in tobacco extracts over 1 g/L, the lethal effect of lower concentrations (0.2 and 0.5 g/L) of tobacco extract on *P. canaliculata* within 15 days was investigated under laboratory conditions in subsequent experiment. Moreover, the effects of tobacco extracts on the behaviour, feeding, growth, antioxidant enzyme and acetylcholinesterase (AChE) activities of *P. canaliculata* were measured. Nine same-sized plastic cases with perforated lids containing 2 L of tobacco extract were used as in the previous experiment for small snails [three replications per concentration (0, 0.2, 0.5 g/L)]. The survival of the snails was checked daily, and their behaviour was observed at 1, 2, 3, 4, 6, 9, 12, and 15 days after ten preweighed small snails were released in each case. The water temperature fluctuated between 28 and 30 °C during the experiment. The individual behaviour of the snails was classified into five states of activity: feeding, crawling, motionless (operculum open), closed operculum and crawling out of water. The behaviour was defined as feeding if the snails eat and crawl simultaneously. Preweighed fresh lettuce was added to each case daily and the leftovers were weighed again after 24 h to estimate the amount ingested by snails, the daily intake per snail was obtained by the lettuce consumption divided by the number of living snails. Dead snails were removed from their respective cases once found, and water was replaced every 2 days. The live snails were weighed again after being released for 15 days to calculate their growth in response to tobacco extracts.

The initial weight (*W*_0_) of individual snails was in agreement with the precision of the scale (0.1 mg) and was obtained by the total weight of the snails in each case divided by the number of living individuals. The snails were allowed to crawl over a plastic surface for a few minutes to expel the water retained in the pallial cavity before weighing^18^. The terminal weight (*W*_t_) was measured in the same way after 15 days. The weight growth rate (WGR) was calculated by Eq. (1), the feeding rate (FR) was calculated by Eq. (2), the food conversion ratio (FCR) was calculated by Eq. (3), and the specific growth rate (SGR) was calculated by Eq. (4):

1 $$\mathrm{WGR }(\mathrm{\%})=\frac{{W}_{t}-{W}_{0}}{{W}_{0}}\times 100$$

2 $$\mathrm{FR}(\mathrm{\%}/\mathrm{d})=\frac{2F}{t({W}_{t}+{W}_{0})}\times 100$$

3 $$\mathrm{FCR}(\mathrm{\%})=\frac{{W}_{t}-{W}_{0}}{F}\times 100$$

4 $$\mathrm{SGR}(\mathrm{\%}/\mathrm{d})=\frac{\mathrm{ln}{W}_{t}-\mathrm{ln}{W}_{0}}{t}\times 100$$

where *t* is 15 days and *F* is the sum of the daily food intake per snail within 15 days.

A similar experiment was conducted for medium snails, ten preweighed individuals were released in each case and nine cases has been used, but the volume of water used was 3 L to prevent the living space from being too small. For adult snails, nine rectangular aquaria (56 × 45 × 35 cm) containing 30 L of tobacco extract (0, 0.2, 0.5 g/L) were used, and ten males and ten females were weighed and introduced into each aquarium. Each aquarium was covered with a 1.5-mm mesh plastic net to prevent the escape of snails. The water temperature was approximately 27.9‒30.2 °C. The feeding and growth of adult snails were investigated in the same way, but mating behaviour was incorporated into the behavioural observations for adult snails based on the experimental design for juvenile snails. All snails were transferred to a − 20 °C refrigerator after weighing and prepared for antioxidant enzyme and AChE activity measurements.

The snails were defrosted, and the hepatopancreas was dissected. Two hepatopancreases from small snails were aggregated into one sample because the tissue from a single hepatopancreas was not sufficient to meet the amount of tissue required for testing. For the other types of *P. canaliculata* (medium, adult male and adult female), one hepatopancreas constituted one sample. The antioxidant enzyme and AChE activities in the hepatopancreas of *P. canaliculata* were measured with four replicates for small, medium and adult female snails based on surplus individuals surviving in 0.2 g/L tobacco extract and with three replicates for adult males, one sample represented a replicate. The hepatopancreas was homogenized with precooled physiological saline (0.7% NaCl, 1:9 w/v) and centrifuged at 3000 rpm and 4 °C for 10 min. Supernatants were used for determination of enzymes. The activities of superoxide dismutase (SOD), catalase (CAT), and AChE and the contents of protein, glutathione (GSH), and malondialdehyde (MDA) in the hepatopancreas of *P. canaliculata* were determined using commercially available assay kits (Jiancheng, Ltd, Nanjing, China) in strict accordance with kit instructions.

### Data analysis

The concentrations of tobacco extracts that killed 50% (LC_50_) of *P. canaliculata* snails were estimated by Karber's method^19^. All the data were analysed using SPSS software version 20.0 for Windows (IBM Inc., U.S.A.). For the *P. canaliculata* survival rate under different tobacco extract concentrations within the same snail type and time, data were tested for normality using the Shapiro‒Wilk test followed by Duncan’s multiple comparison test; arcsine transformation was tried or the nonparametric Kruskal‒Wallis and Nemenyi tests were used if the data failed to meet a normal distribution or equal variance was not assumed. The effects of snail size and sex and tobacco extract concentration on the survival rate of *P. canaliculata* were examined according to repeated-measures ANOVA with time as a within-subject factor, followed by Duncan’s multiple comparison test. The effects of snail size and tobacco extract concentration on snail behaviour were assessed using repeated-measures ANOVA. The multiple comparison for the effect of tobacco extract concentration on food intake of *P. canaliculata* was explored using the same approach as that for survival rate, but the data transformation mode was changed from arcsine to logarithmic. The effects of tobacco extract concentration on weight and other feeding indicators of *P. canaliculata* were investigated using independent samples *t* test after logarithmic transformation. Differences in the antioxidant enzyme and AChE activities of *P. canaliculata* between the control and 0.2 g/L tobacco extract treatments were evaluated by independent samples *t* test, and the Mann‒Whitney *U* test was used if the data were nonnormally distributed. Significance was considered at *P* < 0.05, and high significance was considered at *P* < 0.01.

## Results

### Effects of tobacco extract on the survival of *P. canaliculata*

**Figure 1:**
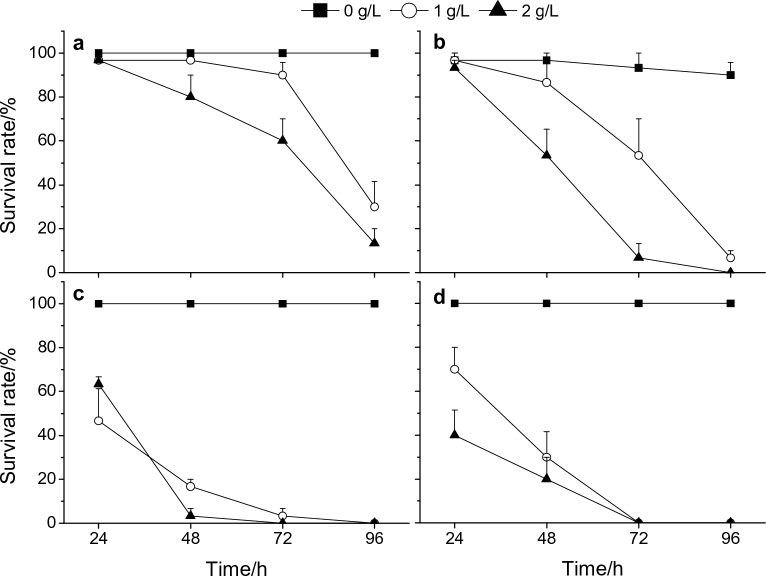
Temporal changes in the survival rate of *P. canaliculata* exposed to various concentrations (0, 1 and 2 g/L) of tobacco extracts within 96 h. 0 g/L tobacco extract (dechlorinated tap water) served as the control group (CK). (**a**–**d**) Small, medium, male adult and female adult of *P. canaliculata*, respectively. Error bars indicate standard error (*n* = 3).

All *P. canaliculata* of different sizes survived at 0 g/L, except for 10% of medium-sized snails that died during the experimental period (Fig. 1; Supplementary Table 1). The survival rates of small snails subjected to various concentrations of tobacco extract were not significantly decreased compared with the control group within 48 h (*P* > 0.05), but the survival rate decreased to 60.0% at 2 g/L tobacco extract after 72 h that were significantly lower than the corresponding control (*P* < 0.05), the survival rates of small snails were 30.0% and 13.3% after exposed to 1 g/L and 2 g/L tobacco extracts for 96 h, respectively (Fig. 1a). Compared with small snails, the medium snails were more sensitive to tobacco extracts and showed lower survival rates at 1‒2 g/L concentrations after 24 h; 6.7% of medium snails survived at 1 g/L tobacco extract, and all died at 2 g/L tobacco extract within 4 days (Fig. 1b). Relative to juveniles (small and medium snails), 1‒2 g/L tobacco extracts caused lower survival rates in adult snails on each investigation day, and all adult snails died under 1 g/L and 2 g/L tobacco extract within 96 h and 72 h, respectively, including males and females (Fig. 1c,d). Specifically, more than 90% of different-sized *P. canaliculata* closed their operculum after exposure to 1‒2 g/L tobacco extract.

The repeated-measures ANOVA revealed marked effects of snail size, tobacco extract concentration, time and their two- and three-dimensional interaction on the survival rates of *P. canaliculata* (regardless of sex; snail size: *F*_2,27_ = 50.024, *P* < 0.001; tobacco extract concentration: *F*_2,27_ = 154.297, *P* < 0.001; time: *F*_3,81_ = 189.455, *P* < 0.001). The size of *P. canaliculata* was inversely proportional to its survival rate in response to the tobacco extracts, and there was a significant difference among the three classes of *P. canaliculata* (*P* < 0.05). Similarly, the higher the concentration of tobacco extract was, the lower the survival rate of *P. canaliculata*, and significant differences were found between different tobacco extract concentrations (*P* < 0.05).

**Figure 2:**
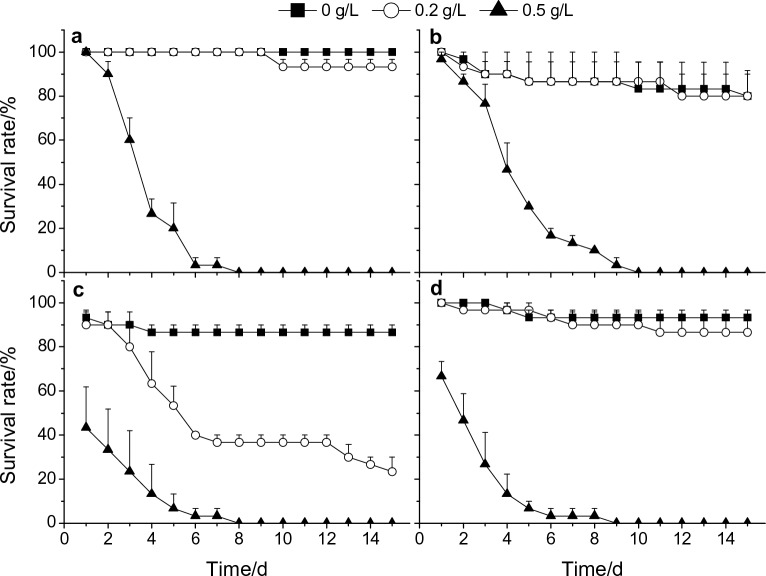
Temporal changes in the survival rate of *P. canaliculata* at low concentrations (0, 0.2 and 0.5 g/L) of tobacco extract within 15 days. 0 g/L tobacco extract (dechlorinated tap water) served as the control group (CK). (**a**–**d**) Small, medium, male adult and female adult of *P. canaliculata*, respectively. Error bars indicate standard error (*n* = 3).

The survival rate of adult male *P. canaliculata* was decreased at 0.2 g/L tobacco extract and fell to 23.3% after 15 days, which was markedly lower than that of the control group from the 5th to 15th day (*P* < 0.05, Fig. 2c). Beyond that, more than 80% of *P. canaliculata* of all sizes survived after exposure to 0.2 g/L tobacco extracts for 15 days, and their survival was not affected compared with the control group (*P* > 0.05, Fig. 2). All snails died within 10 days at a higher concentration of tobacco extract (0.5 g/L), including small, medium, and adult males and females of *P. canaliculata* (Fig. 2; Supplementary Table 2). Repeated measures ANOVA revealed that the survival rate of *P. canaliculata* was inversely proportional to the snail size, tobacco extract concentration and length of exposure time, and these three factors had significant effects on the survival of the snails (*P* < 0.05, Fig. 2). In addition, sex had a significant effect on the survival rate of snails under tobacco stress (excluding juveniles; *F*_1,12_ = 37.538, *P* < 0.001) and female snails had a greater capacity to survive than males after exposure to the tobacco extracts (Fig. 2c,d).

By combining two molluscicidal assay results, the LC_50_ values of tobacco extract for small, medium, adult male and adult female *P. canaliculata* after 48 h of treatment were 0.97, 1.04, 0.51 and 0.55 g/L, respectively. The LC_50_ values of tobacco extract for small, medium, adult male and adult female *P. canaliculata* after 72 h of treatment were 0.80, 0.93, 0.49 and 0.41 g/L, respectively.

### Effects of tobacco extract on the behaviour of *P. canaliculata*

**Figure 3:**
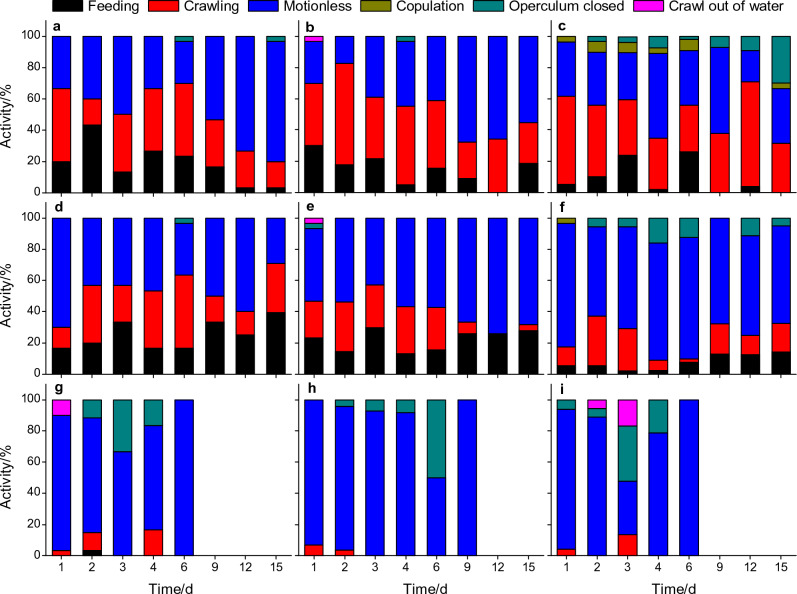
Effect of tobacco extract on snail behaviour. (**a**,**d**,**g**) The activity status of small *P. canaliculata* in 0, 0.2 and 0.5 g/L tobacco extracts, respectively. (**b**,**e**,**h**) The activity status of medium *P. canaliculata* in 0, 0.2 and 0.5 g/L tobacco extracts, respectively. (**c**,**f**,**i**) The activity status of adult *P. canaliculata* in 0, 0.2 and 0.5 g/L tobacco extracts, respectively. There were three replicates for each treatment.

Figure 3 and Supplementary Table 3 shows the behaviours displayed by the different size classes of *P. canaliculata* in the different concentrations of tobacco extract. Significant effects of tobacco extract concentration (*F*_1,12_ = 5.394, *P* < 0.05) and snail size (*F*_2,12_ = 20.261, *P* < 0.001) on feeding behaviour of *P. canaliculata* were observed. Only one small snail exhibited feeding ability in the 0.5 g/L tobacco extract on the 2nd day, and the remaining snails were unable to feed under this condition. In contrast, the snails consumed lettuce at 0‒0.2 g/L tobacco extracts most of the time, but the adult snails showed a lower proportion of feeding behaviour than small and medium snails (*P* < 0.05). The tobacco extracts exhibited a significant effect on the crawling and motionless behaviour of the snails (*P* < 0.01), but the effect of snail size was not significant (*P* > 0.05). With an increase in tobacco extract concentration, more snails reduced crawling and stayed motionless. Few snails displayed the closed operculum behaviour during the experiment and seemed not to be affected by 0.2‒0.5 g/L tobacco extracts (*P* > 0.05), but this phenomenon occurred more commonly in adults. Individual snails crawled out of the water occasionally during the experiment, which was not related to tobacco extract concentration and snail size (*P* > 0.05). Excluding juvenile *P. canaliculata*, the tobacco extracts significantly inhibited the copulation behaviour of the snails (*P* < 0.05). A few snails were found mating in the 0.2 g/L tobacco extract on the 1st day, and no copulatory behaviour was noted in the following observation, including in the snails in the 0.5 g/L tobacco extract.

### Effects of tobacco extract on the feeding and growth of *P. canaliculata*

The snails had difficulty eating food after exposure to 0.5 g/L tobacco extract, and the food intake of different-sized snails was less than 1 g per individual during the experiment, which was much lower than that in the 0 and 0.2 g/L tobacco extracts (*P* < 0.05, Table 1, Supplementary Table 4). However, the amounts of food consumed by small snails were not significantly different between the 0.2 and 0.5 g/L tobacco extracts (*P* > 0.05). Snails of all sizes could obtain food at a lower tobacco extract concentration (0.2 g/L), but their intake was suppressed relative to the control group, except for small snails (*P* < 0.05, Table 1). The WGR, FR, FCR and SGR of small snails were restricted in response to tobacco stress (*P* < 0.05). However, for medium snails, the FR and FCR were not significantly inhibited in the 0.2 g/L tobacco extract group compared with the control group (*P* > 0.05); however, their growth (WGR and SGR) was significantly slowed (*P* < 0.05, Table 1). For adult individuals, their feeding rate was markedly inhibited in 0.2 g/L tobacco extract (*P* < 0.05), but their growth (WGR and SGR) was slightly higher than that of the control group, which may have benefited from their food conversion rate being dramatically increased under the stimulation of tobacco extract (*P* < 0.05, Table 1).

**Table 1:** Effect of tobacco extract on the feeding and growth of *P. canaliculata*.

Snail type	F (g)	W_t_ (g)	WGR (%)	FR (%/d)	FCR (%)	SGR (%/d)
Small						
CK	15.04 ± 0.77^a^	1.96 ± 0.09^a^	192.89 ± 7.19^a^	76.21 ± 1.69^a^	8.59 ± 0.18^a^	7.16 ± 0.17^a^
0.2	11.26 ± 0.20^ab^	1.49 ± 0.05^b^	127.21 ± 7.51^b^	70.01 ± 1.21^b^	7.39 ± 0.24^b^	5.47 ± 0.22^b^
0.5	0.14 ± 0.01^b^					
Medium						
CK	24.02 ± 1.13^a^	3.44 ± 0.13^a^	97.10 ± 14.02^a^	61.64 ± 1.80^a^	6.99 ± 0.46^a^	4.49 ± 0.46^a^
0.2	16.93 ± 2.23^b^	2.87 ± 0.11^b^	56.19 ± 5.89^b^	47.87 ± 5.71^a^	6.15 ± 0.29^a^	2.96 ± 0.25^b^
0.5	0.84 ± 0.30^c^					
Adult						
CK	18.79 ± 0.39^a^	8.92 ± 0.14^b^	6.97 ± 1.00^a^	14.52 ± 0.35^a^	3.09 ± 0.42^b^	0.45 ± 0.06^a^
0.2	8.57 ± 1.30^b^	9.63 ± 0.13^a^	9.32 ± 0.85^a^	6.18 ± 0.89^b^	9.82 ± 0.82^a^	0.59 ± 0.05^a^
0.5	0.60 ± 0.17^c^					

F is the sum of the daily food intake per snail within 15 days. W_t_ is the terminal weight per snail. WGR, FR, FCR and SGR represent weight growth rate, feeding rate, food conversion ratio and specific growth rate, respectively. Different lowercase letters in the same column indicate significant differences between/among treatment groups of *P. canaliculata* of the same type at the 0.05 level.

### Effects of tobacco extract on the antioxidant enzyme and AChE activities of *P. canaliculata*

The antioxidant enzymes (SOD, CAT, GSH and MDA) and AChE activities of small snails and adult females had no significant change after exposure to 0.2 g/L tobacco extract for 15 days compared with the control group (*P* > 0.05, Table 2, Supplementary Table 5). In contrast, all antioxidant indices and AChE activity of adult males were significantly increased in 0.2 g/L tobacco extract (*P* < 0.05). For medium snails, the SOD activity and GSH content were significantly increased under tobacco stress (*P* < 0.05); however, no significant changes in CAT, MDA or AChE activities were observed (*P* > 0.05, Table 2).

**Table 2:** Effect of tobacco extract on the antioxidant enzyme and AChE activities of *P. canaliculata*.

Snail type	SOD (U/mg)	CAT (U/mg)	GSH (mg/g)	MDA (nmol/mg)	AChE (U/mg)
Small					
CK	9.67 ± 0.06^a^	44.47 ± 1.20^a^	3.41 ± 0.51^a^	2.24 ± 0.17^a^	0.16 ± 0.02^a^
0.2	12.63 ± 1.22^a^	50.16 ± 4.54^a^	4.38 ± 0.28^a^	2.46 ± 0.34^a^	0.15 ± 0.01^a^
Medium					
CK	10.27 ± 0.52^b^	36.88 ± 1.93^a^	3.21 ± 0.36^b^	1.71 ± 0.08^a^	0.12 ± 0.01^a^
0.2	12.97 ± 0.66^a^	40.73 ± 2.27^a^	4.33 ± 0.17^a^	2.09 ± 0.30^a^	0.14 ± 0.02^a^
Adult male					
CK	13.93 ± 0.32^b^	32.02 ± 3.46^b^	4.46 ± 0.27^b^	1.40 ± 0.06^b^	0.12 ± 0.00^b^
0.2	16.67 ± 0.80^a^	57.33 ± 1.04^a^	5.58 ± 0.09^a^	1.72 ± 0.05^a^	0.15 ± 0.01^a^
Adult female					
CK	11.76 ± 0.78^a^	30.88 ± 3.34^a^	4.52 ± 0.25^a^	1.43 ± 0.13^a^	0.11 ± 0.02^a^
0.2	12.67 ± 0.63^a^	31.05 ± 2.61^a^	5.28 ± 0.33^a^	1.43 ± 0.16^a^	0.09 ± 0.01^a^

Different lowercase letters in the same column indicate significant differences between treatment groups of *P. canaliculata* of the same type at the 0.05 level.

## Discussion

This study found that *N. tabacum* has great molluscicidal activity against *P. canaliculata*, and the survival rate of *P. canaliculata* decreased with increasing concentration of tobacco extract. The results confirm previous findings that tobacco wastes can be used as potential molluscicides against aquatic snails, and the wastes were not confined to leaves but also included tobacco dust, cigarette processing wastes and smoked filtered cigarette butts^20–23^. Whichever tobacco wastes are used, nicotine is the primary toxicant for controlling snails. A previous study used tobacco waste with 1.9 ppm nicotine at a rate of 1562.5 kg/ha to control *P. canaliculata*^11^. The nicotine content of the tobacco leaves used in this study was 1.1%, which is 5000 times higher than that in the cited study, and nearly 100% of the snails died in 2 g/L tobacco aqueous extract within 4 days. If the water in paddy field keeps at 3 cm deep and 2 g/L tobacco extract is equivalent to 600 kg/ha. Therefore, the amount of tobacco waste used and related labour costs will be greatly reduced. In fact, this study showed that tobacco aqueous extract effectively controlled *P. canaliculata* at 0.5 g/L and superiorly at 1 g/L. If ethanol is used instead of water for extracting tobacco, this will lead to a better inhibitory effect and lower usage^24^. Previous research showed that all apple snails (*P. maculata*) died within 48 h under 0.5 g/L ethanol extract of tobacco leaf, and the LC_50_ was 0.38 g/L^23^. However, aqueous extracts have the advantages of low monetary cost and technological simplicity, and the tobacco waste can be returned directly to the field.

According to our investigation, the lethal effect on *P. canaliculata* under tobacco extract stress was likely inversely linked to individual size because a higher proportion of adult snails died than juveniles (small and medium-sized), moreover, the LC_50_ of adult snails was lower than that of the juveniles and smaller LC_50_ values represented greater molluscicidal activity, which is an unusual observation in *P. canaliculata*, since larger individuals (< 40 mm in shell height) always showed higher survivability either in botanical extracts or chemical molluscicides^3,25,26^. Such a response was consistent with that in nitrogen-fertilized fields^27^. The reason for our discovery might be related to oxygen deficiency; the tobacco organic matter decomposed by microorganisms consumed dissolved oxygen and likely formed an anaerobic environment^28^. In addition, with the increasing concentration of tobacco extract, an oil slick formed that aggravated oxygen consumption. Combined with the fact that oxygen consumption shows a positive relationship with snail size^29^, this may have caused the snails to build up an oxygen debt that worsened with water fouling and poisoning caused by the tobacco extract^30^. Thus, younger snails may be more resistant to tobacco extract, and numerous studies claim that nicotine, as the principal tobacco alkaloid, could be rapidly absorbed by the respiratory system, resulting in cell proliferation and inhibition of apoptosis or even death^31^. However, the lethal effect of oxygen deficiency on *P. canaliculata* in this study still deserves further consideration since a larger space was provided for larger snails. Another possible explanation is that some adult snails might be reaching an advanced age, leading to decreased enzymatic activity and decreased chemical metabolic function^25^. Adults are a high priority in the prevention and control of *P. canaliculata* considering their high food intake and reproductive capacity^17^, and our discovery may be useful for the efficient control of *P. canaliculata*.

In addition to individual size, the sex ratio is another key factor in determining the extent of damage that *P. canaliculata* causes to rice and other aquatic crops^32^. Our study found that females exhibited greater viability than males when exposed to tobacco extract, and may have benefited from their own greater antioxidant capacity. Carotenoids are rich in the albumen gland of female *P. canaliculata* and have strong antioxidant activity^33^, which may reduce the damage caused by tobacco extract to a greater extent than in males. Previous studies have shown that females are more adaptable to cold, heat, drought and fasting than males, and females also grow faster and live longer than males^18,32^. These sex differences indicate that female *P. canaliculata* have higher stress resistance than males. However, from the perspective of survival time, mated females may suffer similar noxious effects of oxygen deficiency as males^30^. In addition, female *P. canaliculata* tend to preserve high energy reserves to achieve higher reproductive success, which is conducive to minimizing the impact of adversity. Males are more susceptible to lethal or sublethal factors under adverse circumstances based on lower energy reserves and higher investment in mate-searching activity^34^.

In addition to the survival of *P. canaliculata*, the behaviour of the snails was significantly affected by tobacco extract. The snails nearly abandoned the chance to feed or mate in 0.5 g/L tobacco extract, and most stayed motionless or with a closed operculum before death; in response, the food intake dropped to below 3.5% of that in the control group. With a reduction in tobacco extract pressure, some snails exhibited feeding and crawling behaviours in 0.2 g/L tobacco extract, but reproduction was still greatly limited such that mating behaviour was observed only on the first day and no spawning occurred during the experimental period. Such a behavioural response was thought to be a strategy for attempting to survive and prosper until favourable conditions reappear^35^, overall, the survival, feeding and reproduction were severely limited under tobacco extract and the extent of inhibition is related to tobacco extract concentration, which indicates that tobacco extract has a marked control effect on the population development of *P. canaliculata*. The snails became inactive under tobacco extract stress likely because they were experiencing double harm from nicotine poisoning and oxygen deficiency. A previous study found that in another freshwater snail, *Melanoides tuberculatus* (Muller 1774), movement gradually decreased with increasing concentrations of tobacco waste, which was probably due to irritation from increased nicotine content^21^. In addition, oxygen restriction negatively affected activity in *P. canaliculata*, and active behaviours could further deplete oxygen^30^.

To survive in water environments containing tobacco waste, aquatic animals consume more energy to prevent physiological damage under inadequate feeding conditions, leading to a reduction in the energy available for growth^36,37^. In this study, the food intake and FR of different-sized *P. canaliculata* dropped to varying degrees under tobacco extract stress, which is possibly a key factor limiting growth performance; in fact, the growth of juvenile *P. canaliculata* was also restricted. Combined with the behaviours displayed by the snails in the tobacco extracts, we speculate that the snails needed energy intake to cope with nicotine poisoning and oxygen deficiency but did not receive sufficient energy to replenish this loss, which may have contributed to their low growth and eventual death^38^. The FCR is inversely proportional to body size in *P. canaliculata*^18^, but the FCR of juvenile snails under tobacco extract stress was below 90% of that in the control group with a smaller size. This phenomenon embodies a trade-off between physiological damage and reduced growth performance. Remarkably, the growth of adult *P. canaliculata* was unaffected by the tobacco extract, driven mainly by an increase in FCR. The potential causes likely included the females maintaining a high survival rate in 0.2 g/L tobacco extract, the females being mildly influenced by low concentrations of tobacco extract based on their strong stress resistance, and even a phenomenon of compensatory growth via increased FCR under tobacco stimulation. Previous research found that *P. canaliculata* snails exhibited compensatory growth by enhancing FR and FCR to protect them from intermittent drought stress^39^. In addition, females are often heavier than males under the same shell height condition^40^, which was reflected in our study. Mass mortality in males under tobacco stress indirectly led to an increase in the average weight of surviving individuals, which increased the feeding and growth data values in a somewhat disguised way.

The hepatopancreas is the key organ of bioaccumulation and detoxification of toxic substances in *P. canaliculata*^41,42^. Various antioxidant enzyme and AChE activity responses in the hepatopancreas have been observed under tobacco extract. Nicotine promotes reactive oxygen species (ROS) generation in a dose-dependent manner^43^, and SOD and CAT are dedicated to removing damaging ROS and are considered the first line of defence against lipid peroxidation and oxidative stress^44,45^. We found that SOD activity was increased by tobacco extract in medium and adult male snails, while a marked increase in CAT activity only occurred in adult males. GSH plays a role as a ROS scavenger and substrate in the antioxidant enzyme system^44^, and the elevation of GSH content in medium and adult male snails was found, which might help them reduce oxidative damage caused by tobacco extract. MDA is the metabolic product of lipid peroxidation and is regarded as a diagnostic indicator of oxidative tissue damage^5^. An increased MDA content was found in adult male *P. canaliculata* after exposure to tobacco extract, which means that the hepatopancreas tissue suffered oxidative damage or physiological injury and may further contribute to snail death. AChE is the primary neurotransmitter in the nervous system of animals and functions by hydrolyzing Ach^5^. In this study, AChE activity was significantly increased by tobacco extract in adult male *P. canaliculata*, and a similar phenomenon has been observed in rats, which may be related to the increase in ACh receptor ligands under nicotine stimulation^43,46^. ACh may be hydrolysed faster by excessive AChE activity^5^, and ACh receptor occupancy by nicotine results in improper ACh conduction^9^, leading to nervous dysfunction and blocked muscle movement, which might contribute to snail death^5^. Synthesizing the above results, it appeared that the responses of these typical antioxidases are size- and sex-dependent, and the antioxidant system of small and adult female *P. canaliculata* might not be activated under low concentrations (0.2 g/L) of tobacco extract, which is consistent with the survival of the snails and the survival advantages of these snails in response to tobacco exposure as described above.

This study comprehensively analysed the molluscicidal activities of tobacco extracts on *P. canaliculata* by measuring the survival, behaviour, feeding, growth, antioxidant enzyme and AChE activities of the snails, which indicated that discarded tobacco leaf has a strong molluscicidal activity and may have potential for use in the population management of *P. canaliculata*. Nevertheless, further field work is warranted to confirm the molluscicidal activity of tobacco extract on *P. canaliculata* in the context of complex ecosystems and to determine whether the lethal and sublethal effects of tobacco extract will be weakened by soil or other components in the field. The molluscicidal activities of extracts from different parts of discarded tobacco on *P. canaliculata* and the effects on rice or other crops also need to be further evaluated. It is remarkable that 1 g/L powdered tobacco leaves caused high mortality (> 80%) to adult *P. canaliculata* after introduced to simulated paddy field within 96 h, and promoted the growth of rice seedlings. But some snails could nibble on rice seedlings under 0.5 g/L powdered tobacco leaves. It's probably better approach that around 1 g/L powdered tobacco leaves is introduced to rice field 2‒4 days before transplanting based on our experience, but needs further research and summarization.

## Conclusions

Our results showed that tobacco extract induced acute toxicity to *P. canaliculata* snails over 1 g/L, and molluscicidal activity progressively increased with the extract concentration and exposure time. Regardless of sex, the survival rate of *P. canaliculata* under tobacco extract stress was inversely proportional to its size, and adult males were more susceptible to the tobacco extract than females. The snails nearly abandoned the chance to feed or mate in 0.5 g/L tobacco extract, and reproduction was greatly limited at low concentrations (0.2 g/L). Long-term exposure to 0.2 g/L tobacco extract inhibited food intake in larger snails (> 15 mm shell height) and limited growth in juveniles (< 25 mm shell height). The antioxidant enzyme and AChE activities in the hepatopancreas of *P. canaliculata* in response to tobacco extract were size- and sex-dependent. The activities of SOD, CAT, AChE and the contents of GSH and MDA were increased in adult males after exposure to tobacco extract for 15 days, and increased SOD activity and GSH content were found in medium-sized snails (15‒25 mm shell height). These results suggest that discarded tobacco leaves can be useful as a molluscicide for controlling the invasive snail *P. canaliculata* based on its effects on survival, behaviour, food intake, growth performance and antioxidant capacity.

## Supplementary Information

Supplementary Table 1.

Supplementary Table 2.

Supplementary Table 3.

Supplementary Table 4.

Supplementary Table 5.

## Data Availability

The datasets used and/or analyzed during the current study are included in this published article [and its supplementary information files].
